# Secondary Hemochromatosis Leading to Acute Coronary Syndrome in a Thalassemic Patient

**DOI:** 10.7759/cureus.48226

**Published:** 2023-11-03

**Authors:** Hidayat Ullah, Noman Salih, Ani Tavaratsyan, Maharshikumar Sandesara, Sarah Syed, Najam Us Saher, Tamara Fleihan

**Affiliations:** 1 General Practice, Hayatabad Medical Complex Peshawar, Peshawar, PAK; 2 Internal Medicine, Hayatabad Medical Complex Peshawar, Peshawar, PAK; 3 Cardiology, Yerevan State Medical University (YSMU), Yerevan, ARM; 4 Medicine, C.U. Shah Medical College and Hospital, Surendranagar, IND; 5 Medicine, Combined Military Hospital (CMH) Lahore Medical College and Institute of Dentistry, Lahore, PAK; 6 Neuroscience, Baylor College of Medicine, Houston, USA

**Keywords:** troponin levels, acute coronary syndrome, erythropoiesis, cardiac dysfunction, thalassemia major

## Abstract

Thalassemia, a congenital hemoglobinopathy, is characterized by impaired erythropoiesis and peripheral hemolysis, leading to anemia. Thalassemia major, in particular, necessitates regular blood transfusions, resulting in iron accumulation in the body. Iron overload primarily affects the heart and can induce cardiac disorder, including defects in the pump and conduction system, which is one of the leading causes of mortality among thalassemics. The existing literature has revealed limited support for the occurrence of acute coronary syndrome (ACS) due to hemochromatosis. However, it does show that elevated troponin levels can be observed even in cases not associated with ACS. Here, we offer a rare case study of acute coronary syndrome in a patient with thalassemia major who also had elevated ferritin levels and abnormal troponin I values. The difficulty of cardiac problems in thalassemia major is highlighted by this case, as well as the necessity for more clinical attention and study to better comprehend and handle such instances.

## Introduction

Iron is a crucial component required for a number of biological and metabolic functions. However, an excessive amount of iron in the body can lead to oxidative stress, which can harm tissues [1]. This additional iron tends to accumulate throughout the body, especially in the liver, spleen, heart, bones, pancreas, and nervous system, potentially hurting these important organs.

One of the most severe outcomes of iron overload is the emergence of iron excess-induced acute coronary syndrome (ACS), as well as iron overload cardiomyopathy (IOC), which is brought on by the accumulation of iron in the myocardium. Patients who require continuous blood transfusion therapy frequently die from IOC and other cardiac complications [2]. The global occurrence of IOC and ACS associated with iron overload is increasing, with cardiologists typically overseeing treatment. It is important to highlight that IOC is becoming more prevalent in patients with hematologic malignancies, particularly with the increasing frequency of therapies such as bone marrow transplantation and stem cell transplantation [3]. Furthermore, as persons with illnesses such as thalassemia as well as sickle cell disease live longer, the number of cases of ACS is also increasing. It is generally known that if cardiac abnormalities are detected before they advance to end-stage heart conditions, early detection and proper medical attention can reverse these conditions [4], emphasizing the significance of early detection. While there is enough information available about other cardiac complications such as IOC, there is limited information available on iron-induced ACS.

Therefore, it is of utmost importance for healthcare providers specializing in cardiology and internal medicine to continuously update their knowledge regarding ACS management, benefiting from recent advancements in this field. This article aims to present an overview of a unique case involving non-ST-elevation myocardial infarction (NSTEMI) induced by secondary hemochromatosis.

## Case presentation

A 20-year-old female patient with an earlier diagnosis of thalassemia major, which had necessitated over 800 transfusions, presented to our emergency department due to sudden and severe chest discomfort. The left side of the chest was the specific location of the discomfort, which spread to her jaw and was accompanied by other symptoms including a severe headache plus shortness of breath. The pain was sudden in onset with rapid progression, squeezing in nature, and not relieving with over-the-counter pain medication. Despite trying over-the-counter painkillers at home, the pain persisted, prompting her hospitalization. She was on chelation therapy, but her chelation therapy had not been properly adjusted.

Upon assessment, the patient's temperature was 99°F (37.23°C), her heart rate was 94 beats per minute, her respiratory rate was 21 breaths per minute, and her blood pressure was 115/75 mmHg. Physical examination revealed darkened skin and a slightly pale conjunctiva. The abdomen was soft but slightly distended, with the liver palpable about 2 inches below the right ribcage, while her spleen was not palpable. There were no signs of jaundice, and a chest examination revealed clear breath sounds. No pitting edema was observed in her legs, and her neurological assessment yielded no abnormalities.

**Figure 1:**
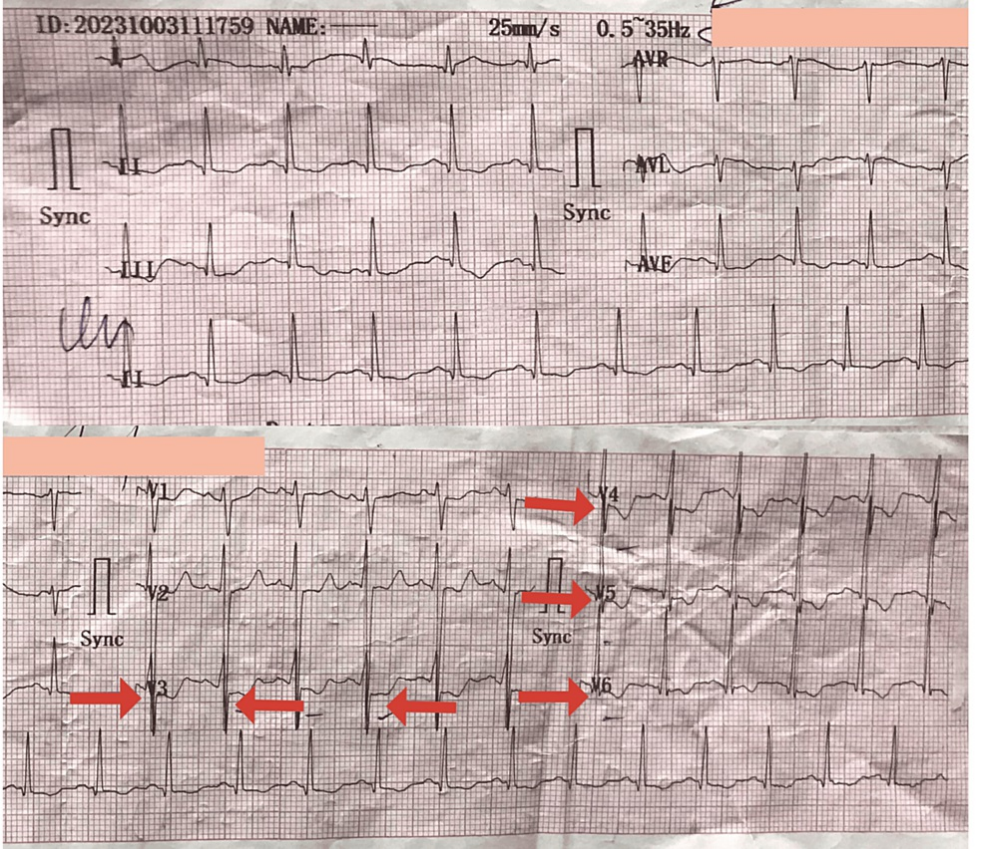
ST-segment depression in chest leads V3, V4, V5, and V6 (red arrows)

An electrocardiogram (ECG) shown in Figure 1 indicated a non-ST-elevation myocardial infarction (NSTEMI), which was further corroborated by significantly elevated cardiac enzyme levels. Laboratory investigations shown in Table 1 revealed microcytic anemia, characterized by low hemoglobin and mean cell volume. Ferritin levels were elevated, consistent with her medical history, while other laboratory results remained within normal limits. Abdominal and pelvic ultrasound examinations ruled out gastrointestinal causes for chest pain, confirmed the absence of a spleen, and suggested deposits in the liver. Additional chest ultrasound revealed cardiac enhancement, prompting a recommendation for further confirmation through either a CT scan or MRI. An echocardiogram showed no abnormalities, and chest radiography did not point out any significant findings.

**Table 1:** Laboratory investigations Abbreviations: ALP, alkaline phosphatase; ALT, alanine aminotransferase; AST, aspartate aminotransferase; BUN, blood urea nitrogen; Creat, creatinine; WBC, white blood cells; Hb, hemoglobin; MCV, mean cell volume; Na, sodium; K, potassium; RBS, random blood sugar; LDL, low-density lipoprotein; TAG; triacylglycerol

Test	Reference value	Patient's value
WBC (/µL)	4,000-11,000	10,500
Hb (g/dL)	11.5-17.5	8.9
MCV (fL)	80-100	67.8
Platelets (/µL)	150,000-450,000	165,000
BUN (mg/dL)	18-45	39
Creat (mg/dL)	0.3-0.9	0.59
ALT (U/L)	10-50	73
AST (U/L)	8-33	56
ALP (U/L)	<390	357
Bilirubin (mg/dL)	0.1-1	1.2
Na (mmol/L)	135-150	133.9
K (mmol/L)	3.5-5	3.95
Ferritin (ng/mL)	12-150	5,871
Transferrin saturation (%)	20-50	85
RBS (mg/dL)	100-125	145
Troponin I (ng/mL)	0-0.04	12.09
LDL cholesterol (mg/dL)	<100	48
TAG (mg/dL)	<150	134

**Figure 2:**
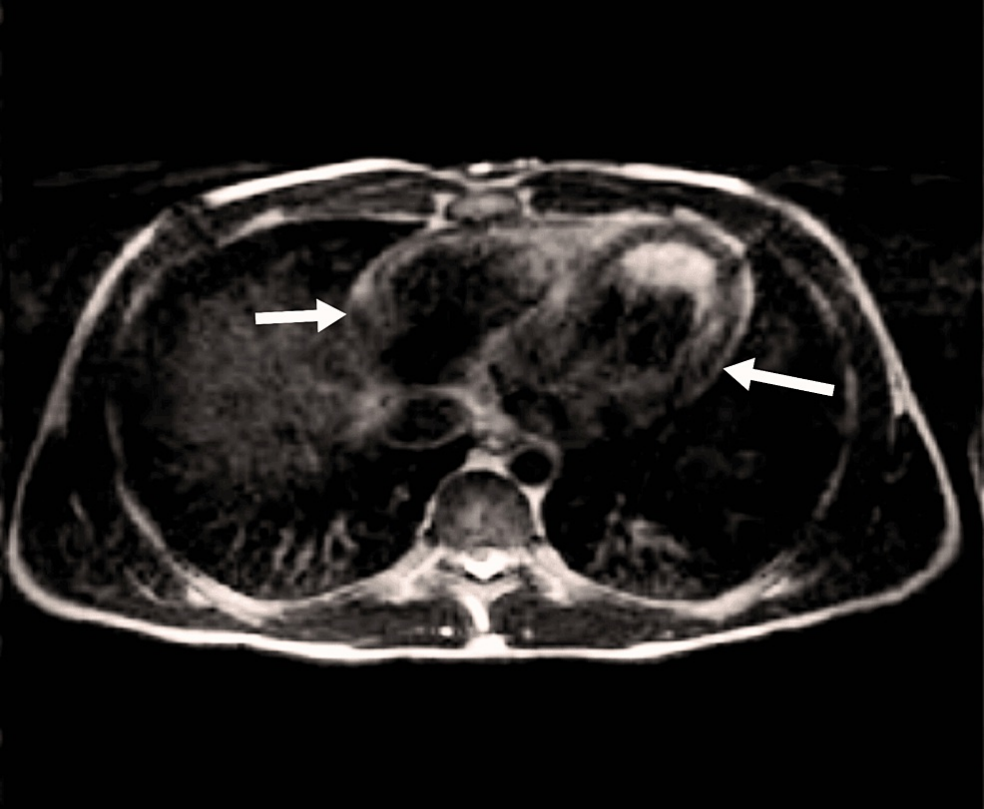
Iron deposits in the myocardium (white arrows)

Given the heightened sensitivity and specificity of MRI for detecting iron deposits, an MRI of the pelvis, abdomen, and chest was done, demonstrating widespread low signals in the heart, pancreas, and liver as shown in Figure 2. Patient management included the administration of aspirin, beta-blockers, and clopidogrel as a precautionary measure, despite their limited role in this case.

As the patient did not initially present within the window for coronary angiography, a delayed angiography was performed, which revealed normal coronary vessels. The patient had been on specialized iron-chelating medication (deferasirox 1,500 mg/day) for 13 years to manage elevated blood ferritin levels. The chelation therapy dosage was increased to eliminate the remaining iron and prevent future acute coronary syndrome attacks. The patient was advised to make necessary life changes such as following heart-healthy diets, doing regular exercise, and not taking too much stress.

During a follow-up visit two weeks after discharge, the patient's symptoms had improved. She was advised to continue her chelation therapy and return promptly if her previous symptoms reappeared or if new symptoms arose.

## Discussion

Chronic blood transfusions are known to cause the well-known complication of iron overload in people with thalassemia major, known as iron overload cardiomyopathy (IOC) [5,6]. However, the occurrence of acute coronary syndrome (ACS) such as non-ST-elevation myocardial infarction (NSTEMI) as in our patient in the context of thalassemia major, especially with abnormal troponin I level, is rare and poses a unique clinical challenge.

Iron accumulation in various organs is a hallmark of thalassemia major, potentially affecting the heart, liver, spleen, and other vital organs [7]. The heart is particularly vulnerable to damage brought on by iron overload, and IOC is a major cause of morbidity and death in these individuals [8].

This case report presents a 20-year-old patient with thalassemia major who experienced ACS along with elevated troponin I levels. The patient had a history of over 800 transfusions, leading to iron overload and subsequent cardiac complications [9]. Despite being on chelation therapy, her therapy had not been adequately adjusted, leading to persistent iron overload.

The clinical presentation included sudden and severe chest pain, indicative of ACS [10]. Initial examinations revealed physical signs such as darkened skin, microcytic anemia, and hepatomegaly. An electrocardiogram (ECG) showed non-ST-elevation myocardial infarction (NSTEMI), which was further corroborated by considerably raised cardiac enzyme levels.

Further investigations, including imaging studies, were conducted to assess the extent of iron deposition and cardiac involvement [11]. Abdominal and pelvic ultrasound examinations confirmed iron deposition in the liver but ruled out gastrointestinal causes for chest pain. Importantly, cardiac enhancement was detected on chest ultrasound, prompting further evaluation through MRI, which revealed diffuse low signals in the liver, pancreas, axial skeleton, and heart [7,12].

Although the patient did not initially present within the window for coronary angiography, a delayed angiography was performed, revealing normal coronary vessels. The patient had been on specialized iron-chelating medication (deferasirox 1,500 mg/day) for eight years to manage elevated blood ferritin levels [13-15]. The chelation therapy dosage was increased to eliminate the remaining iron and prevent future ACS attacks.

## Conclusions

With an emphasis on the probable incidence of iron-induced acute coronary syndrome (ACS) coupled with abnormal troponin levels, this case report highlights the complexity of cardiac problems in people with thalassemia major. Patients with thalassemia major who have iron overload must have prompt diagnosis and careful therapy in order to reduce their chance of developing ACS, underscoring the critical need for healthcare provider's ongoing attention. Furthermore, continuous research initiatives are crucial because they advance our knowledge of these complex clinical situations. In the long run, this increased body of information will help in the efficient management of thalassemia major, resulting in better patient outcomes and a greater standard of living for thalassemic patients.
